# Paraneoplastic pemphigus and myasthenia gravis as the first manifestations of a rare case of pancreatic follicular dendritic cell sarcoma: CT findings and review of literature

**DOI:** 10.1186/s12876-019-1008-y

**Published:** 2019-06-14

**Authors:** Tao Lu, Bin Song, Hong Pu, Xinglan Li, Qiqi Chen, Chong Yang

**Affiliations:** 1 Department of Radiology, West China Hospital, Sichuan University, No. 37, Guoxuexiang, Chengdu, 610037 Sichuan China; 2 Department of Radiology, Sichuan Academy of Medical Science and Sichuan Provincial People’s Hospital, 32 West Second Section, First Ring Road, Chengdu, 610072 Sichuan China; 3 Department of Pathology, Sichuan Academy of Medical Science and Sichuan Provincial People’s Hospital, 32 West Second Section, First Ring Road, Chengdu, 610072 Sichuan China; 4 Department of Rheumatology, Sichuan Academy of Medical Science and Sichuan Provincial People’s Hospital, 32 West Second Section, First Ring Road, Chengdu, 610072 Sichuan China; 5 Department of Organ transplantation, Sichuan Academy of Medical Science and Sichuan Provincial People’s Hospital, 32 West Second Section, First Ring Road, Chengdu, 610072 Sichuan China

**Keywords:** Follicular dendritic cell sarcoma CT paraneoplastic pemphigus myasthenia gravis

## Abstract

**Background:**

Follicular dendritic cell sarcoma (FDCS) is a rare neoplasm that originates from follicular dendritic cells in lymphoid tissue while paraneoplastic pemphigus (PNP) is an autoimmune blistering disease associated with neoplasms. Pancreatic FDCS associated with PNP and myasthenia gravis (MG) is even rarer and highly malignant. We present the clinical data, pathological materials and computed tomography (CT) features of a rare case of this disease.

**Case presentation:**

A 49-year-old woman presented with repeated ptosis of both eyelids, oral ulcers and erosions. Her laboratory results showed a slight elevation of CA125 and positivity of some autoimmune antibodies. CT revealed a round solid mass with central necrosis in the pancreatic tail. The solid component of the mass showed slight enhancement and serpentine feeding arteries in the arterial phase, moderate enhancement with a draining vein around the tumor in the portal venous phase and persistent enhancement in the delayed phase. Surgical resection was performed, and the pathological diagnosis was FDCS. However, the patient died of inability to excrete sputum and occlusion of the respiratory tract.

**Conclusions:**

Pancreatic FDCS manifested as PNP and MG is very rare. Its CT features are not specific, and the disease should be differentiated from neuroendocrine tumors, solid pseudopapillary neoplasms and acinar cell carcinoma.

## Background

Follicular dendritic cells (FDC), also recognized as dendritic reticulum cells, are an indispensable part of B-cell follicles. Their functions include antigen presentation, generation and regulation of the germinal center reaction [1]. Follicular dendritic cell sarcoma (FDCS) was first recognized in 1986 by Monda et al., and is a rare neoplasm that originates from these follicular dendritic cells [2]. Since then, more than 300 patients of FDCS have been reported worldwide [3]. Approximately 2/3 of the cases were found in lymph nodes, particularly in the cervical lymph nodes and occasionally in the axillary and mediastinal lymph nodes, whereas 1/3 of the cases were found in extranodal sites, especially in the abdominal cavity and pelvic region, then in the neck and chest, and rarely in the breast, thigh, groin, dura mater encephali and skin [4].

The abdominal cavity is a more desirable site of extranodal FDCS with a preference of the pancreas and peripancreatic tissues [1, 4]. To our knowledge, to date, only 5 cases of pancreatic FDCS have been reported [5–8], and none of the reported cases manifested as paraneoplastic pemphigus and myasthenia gravis. We herein report a case of pancreatic FDCS with paraneoplastic pemphigus and myasthenia gravis as the first manifestations and describe the CT features of the tumor.

## Case presentation

**Fig. 1:**
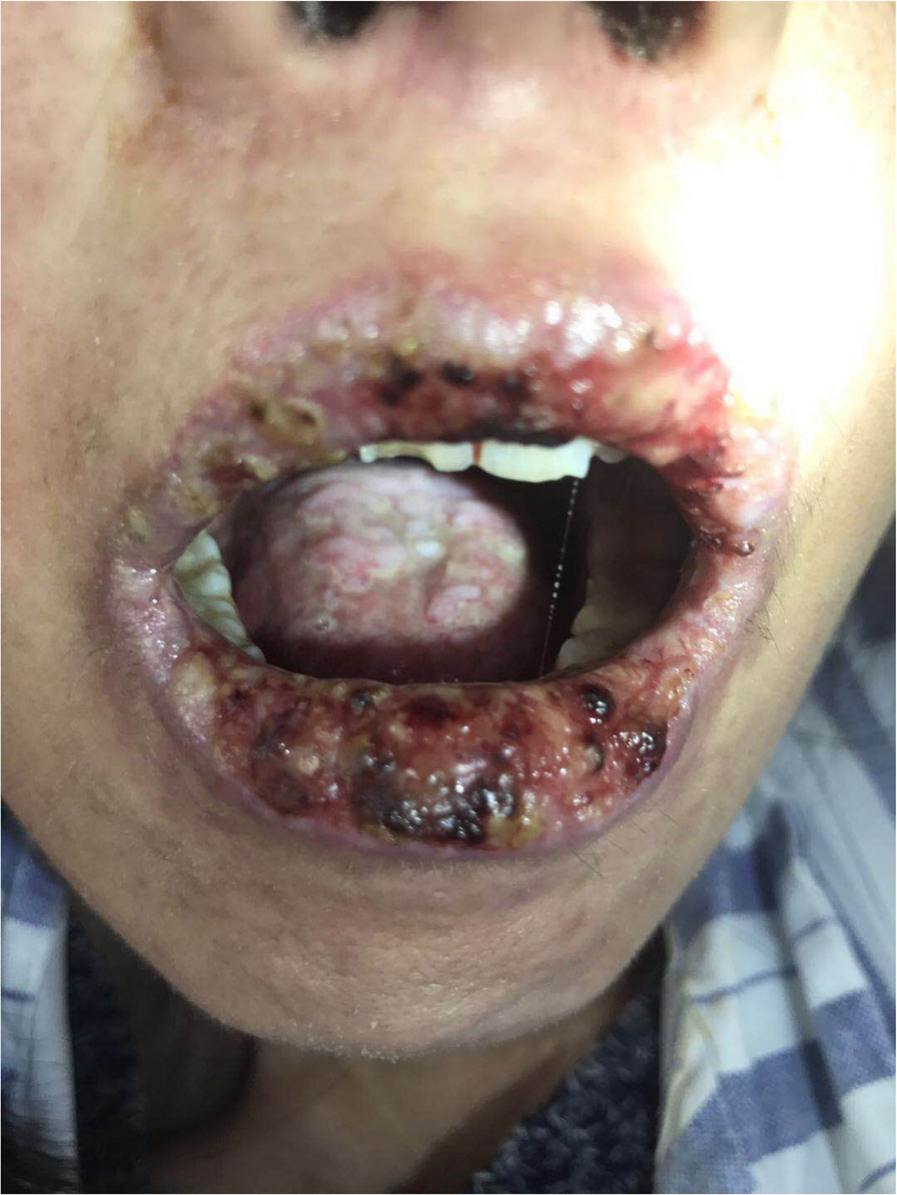
Scattered ulcers and erosions in the mouth of the patient

A 49-year-old woman presented with a 3-month history of repeated ptosis of both eyelids and oral ulcers and erosions. Physical examinations revealed scattered ulcers and erosions in the mouth (Fig. 1). Laboratory examinations showed that CA 125 was elevated (51.6 U/ml), while other tumor markers, including CA199, 153, CEA, and AFP, were normal. Autoimmune antibodies, including anti-CENP-B antibody, ANCA, anti-AchR antibody, and ANA, were all positive.

**Fig. 2:**
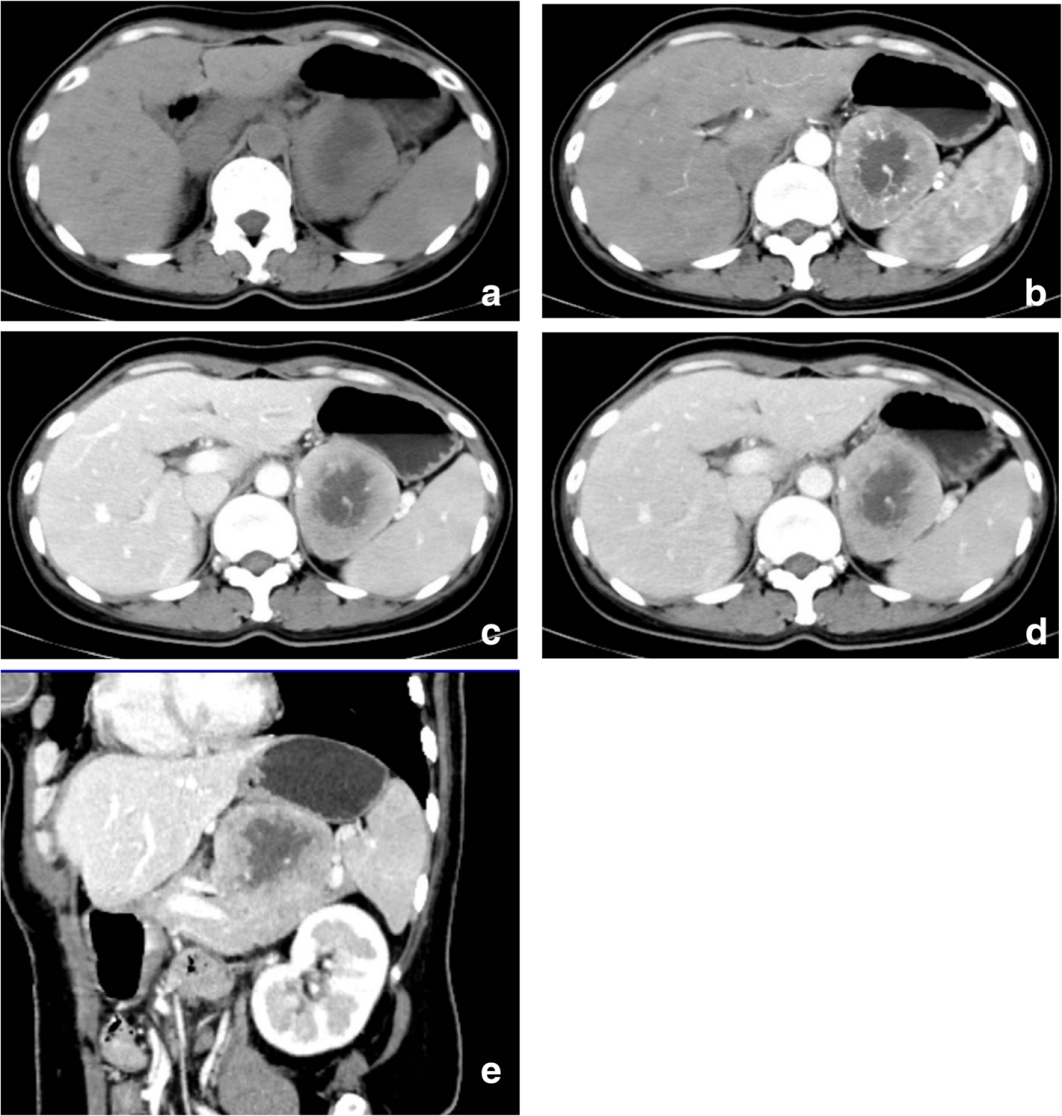
**a**-**e** A well-defined round solid mass with central necrosis in the pancreatic tail

The patient was first diagnosed with an oral aphthous ulcer and ocular myopathy myasthenia gravis. She was treated with gentamycin and dexamethasone spray inhalation to improve her oral lesions and pyridostigmine to cure muscle weakness. However, the oral ulcers improved slightly and the myasthenia gravis persisted. An abdominal ultrasound showed a hypoechoic mass in the left adrenal gland. A further CT examination showed a 6 × 5 cm, well-defined round solid mass with central necrosis in the pancreatic tail. There was no calcification detected in the mass. The solid part of the mass had slight enhancement in the arterial phase with many serpentine feeding arteries, moderate enhancement with a draining vein around the tumor in the portal venous phase and persistent enhancement in the delayed phase (Fig. 2a-e). The fundus of the stomach was compressed by the mass. The boundary between the mass and the splenic artery and vein was not clear, and swollen lymph nodes were not observed in the posterior peritoneum. The mass was initially considered to be a neuroendocrine tumor in the pancreatic tail.

At the same time, the patient’s symptoms worsened. She could not swallow, and she felt severe pain in her mouth. She also developed a cough and expectoration. A chest CT revealed infection in the lower lobes of both lungs. Streptococcus was detected from a throat swab. Levofloxacin was administered to fight the infection, methylprednisolone to fight the inflammation, and thalidomide to alleviate the vascular inflammatory reaction in addition to pyridostigmine and immunomodulatory therapy. However, 3 days later, the patient progressed to severe dyspnea, wheezing and difficulty with expectoration. Emergency intubation and mechanical ventilation were administered. Aspergillus was detected after bronchoalveolar lavage. Immunoglobin and voriconazole were given. Four days later, the symptoms resolved and the intubation was detached. Most of the infections in the lungs were resolved according to a chest CT. The pain in the mouth was also alleviated.

**Fig. 3:**
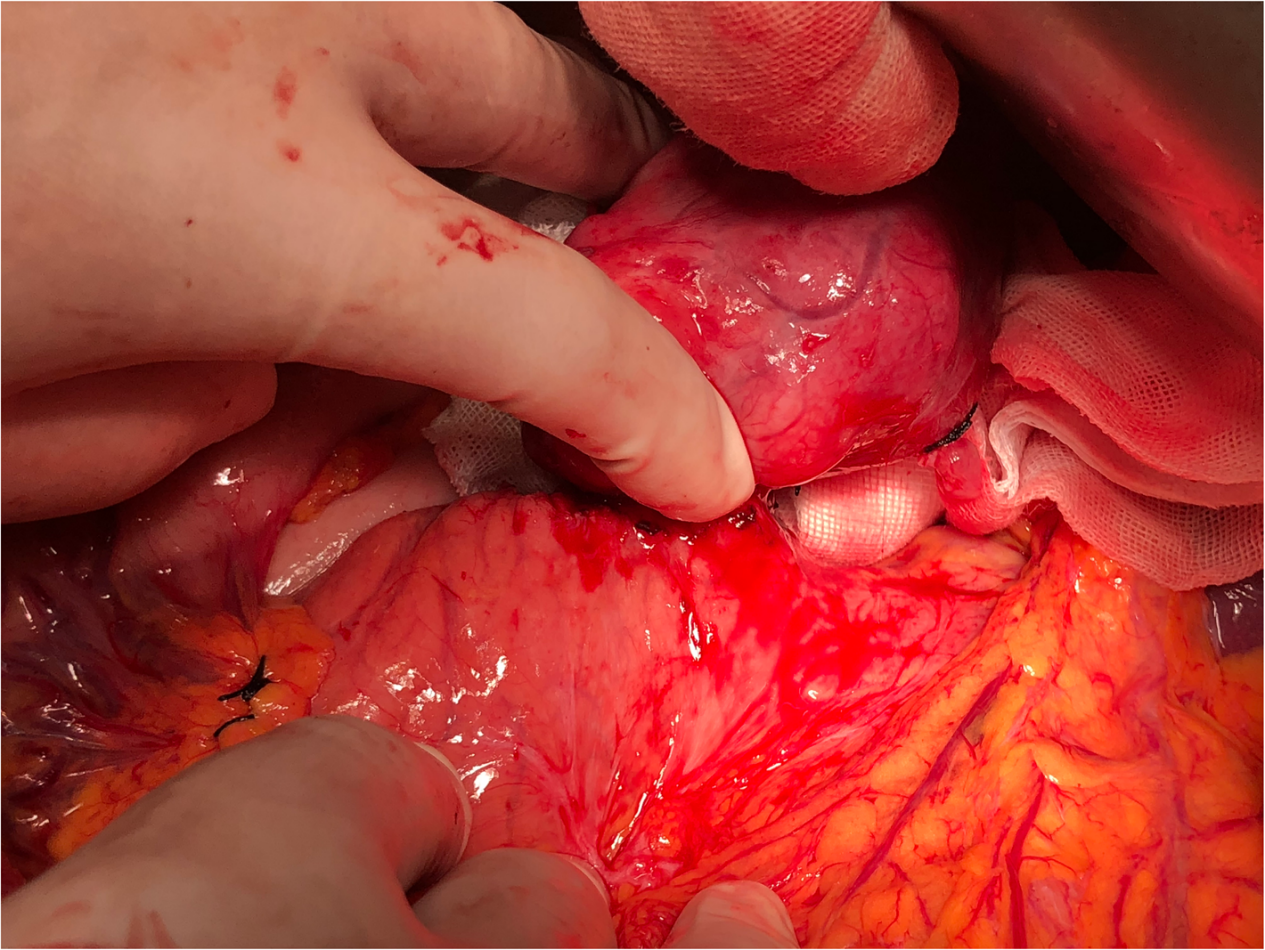
Dark-red tumor in the pancreatic tail during surgery

**Fig. 4:**
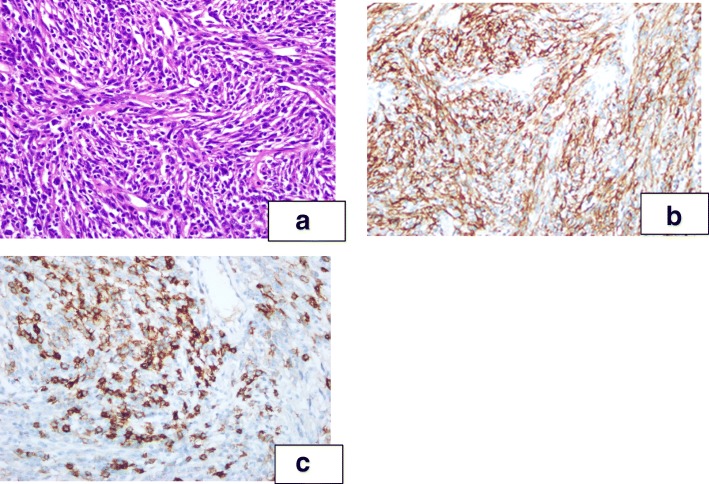
**a**-**c** Microphotograph(**a**) showing spindle tumor cells(×200). Immunohistochemistry of the tumor showing positivity for CD21 (**b**) and CD23(**c**) (×200)

After a multidisciplinary discussion, the patient’s tentative diagnosis was paraneoplastic pemphigus and the myasthenia symptoms caused by the pancreatic tumor. Myasthenia gravis, in turn, led to the patient’s inability to excrete sputum. If the pancreatic tumor could not be removed, the symptoms would not completely remit, and the symptoms due to myasthenia gravis would also continue to aggravate, finally leading to the occlusion of the respiratory tract. Therefore, the patient was transferred to general surgery. During the surgery, a 4 × 5 cm dark-red tumor with medium texture and clear boundaries was detected in the pancreatic tail (Fig. 3). The tumor was close to the splenic artery and vein, and the spleen was normal. Postoperative pathology confirmed the tumor was a follicular dendritic cell sarcoma with immunohistochemistry showing CD21(+), CD23(+), CD138(+), SMA(+), Des(+), CD117(−), DOG-1(−), S-100(−), CD34(−), CK(−), EBER and EBV(−)(Fig. 4a-c).

The patient continued to be treated with antifungal and anti-infection therapy. Twelve days after surgery, the patient developed sudden heart palpitation, discomfort and difficulty in breathing. Mechanical ventilation was again administered. However, the patient died of inability to excrete sputum and occlusion of the respiratory tract.

## Discussion and conclusions

Extranodal FDCS was first reported by Chan et al. in 1994 [9]. After that, a great spectrum of FDCS in extranodal sites has been reported, including the tonsils, pharyngeal region, thyroid, mediastinum, intra-abdominal sites and retroperitoneum [10, 11]. Extranodal FDCS can ocurr between 9 and 82 years of age with an average age of onset in the fourth decade, and has a slight female predominance [4, 11].

A review of the literature suggests only 5 cases of pancreatic FDCS have been previously realized. In the 5 cases, the age of the patients ranged from 56 to 70 years, and there was an obvious predilection for men, with a female to male ratio of 1:4. The occurrence was in the pancreatic head in 3 cases and in the tail in 1 case, and the authors did not specify the location of the tumor in the remaining 1 case. The maximum diameter of the tumor ranged from 2 to 11 cm in 4 cases, and the authors did not specify the exact size of the tumor in 1 case. In our case, the pancreatic tumor occurred in the tail in a 49-year-old woman with a maximum diameter of 5 cm. In 2 previous reports, the clinical presentations of pancreatic FDCS included weight loss, poor appetite, nausea, abdominal fullness and mass, in another report, the patient had no symptoms at all, and in the remaining 2 reports, the authors did not describe the patients’ symptoms. In our case, the patient presented with paraneoplastic pemphigus and myasthenia gravis. There are few reports of intra-abdominal FDCS manifested as paraneoplastic pemphigus. In addition, our case is the only case of pancreatic FDCS with the initial presentation as paraneoplastic pemphigus and myasthenia gravis.

FDCS has traditionally been considered as an indolent tumor and has a tendency of local recurrence and a low risk of metastases. Chan et al. [1] analyzed the clinicopathologic features of 17 cases of FDCS and found that the overall recurrence, metastasis and mortality rates were 43, 21 and 17%, respectively. FDCS should be considered to be a tumor with at least intermediate grade malignancy. It has been reported that abdominal FDCS tends to manifest as larger-sized tumors than those outside the abdomen with the average tumor size of 10.2 cm (range 3–22 cm) [11]. An intra-abdominal location was also considered to be the single most important unfavorable prognostic factor for FDCS [12]. Extrinsic necrosis, a larger tumor size (> 6 cm), cytologic atypia and a high proliferative index (mitotic count > 5 mitoses/10 high-power fields) are other poor prognostic factors, all of which increase the likelihood of recurrence, metastasis and death [10].

The gold standard for the diagnosis of FDCS is the histologic appearance and specific immunohistochemical staining pattern. Proliferation of spindle-to-ovoid shaped cells arranged in storiform, fascicular and whorled patterns with a sprinkling of small lymphocytes throughout the tumor and perivascular lymphocytic cuffing are the main histologic features of FDCS [1]. Ultrastructure studies are desirable but not essential. CD21, CD35 and CD23 are the most widely used FDCS markers. It can also be variably positive with other markers, including S-100, EMA, CD68, CD45, CD3, CD20, HMB-45 and Ki-67. The immunophenotypic profile in our case conformed to that of previous reports.

Abdominal FDCS has been described as a well-defined mass with central necrosis and/or coarse internal calcification, homogeneous or heterogeneous enhancement and regional lymphadenopathy [13, 14]. Li et al. [14] reported that abdominal FDCS showed marked or moderate enhancement with ‘rapid wash-in and slow wash-out’ or ‘progressive enhancement’ patterns during multiphase imaging with or without serpentine feeding arteries and draining veins in and/or around the tumor. For the previous published 5 cases of pancreatic FDCS, only 2 described the imaging features of the tumors. One tumor was heterogeneous in the pancreatic head with dilation of the biliary tree, the other tumor was a clear-defined, round, solid mass with a cystic component in the pancreatic tail with slight contrast enhancement. The tumor in our case was also in the pancreatic tail. It was a round solid mass with a central cystic area but without calcification. The margin of the tumor was also clear. After enhancement, the solid component of the tumor demonstrated a ‘progressive enhancement’ pattern with many serpentine vessels detected in the arterial phase and a draining vein around the tumor in the venous phase. Since the tumor was in the pancreatic tail, it was impossible for it to result in dilation of the bile duct and pancreatic duct. The enhancement pattern in our case suggested that the tumor was relatively hypervascular, which was similar to some previous reports [12, 13]. Second, the ‘progressive enhancement’ in the venous and delayed phases are thought to correlate with hypercellular density areas with proliferation of spindle to ovoid cells and to be associated with lymphocyte-rich stroma [13]. The presence of a cystic area in the tumor may be related to the tumor size, as a large tumor is prone to degeneration or necrosis. Some enlarged lymph nodes were detected in the peripancreatic area or in the retroperitoneum in the previous 2 reports of pancreatic FDCS. However, in our case, lymphadenopathy was not detected.

From imaging, the differential diagnosis of pancreatic FDCS include neuroendocrine tumors, solid pseudopapillary neoplasms and acinar cell carcinoma. Pancreatic neuroendocrine tumors are usually hypervascular with areas of necrosis and cystic degeneration in larger tumors. Large, non-hyperfunctioning endocrine tumors usually have cystic changes, and the patients with those tumors also present late due to the lack of endocrine symptoms [14]. Typically, the tumors enhanced rapidly, with the thick cyst walls enhanced with an irregular inner surface [15]. The pancreatic FDCS in our case was a solid mass with a central cystic area, but it showed a progressive enhancement pattern, and the inner surface of the cyst wall was smooth. Solid pseudopapillary tumors (SPT) are commonly seen in young females without symptoms or can be with vague abdominal pain, dyspepsia and bloating [16]. The tumor typically located in the tail of the pancreas with internal architecture. Hemorrhage or necrosis of the tumors can be easily discriminated on CT. A solid part with contrast enhancement and calcification on the capsule may also be present. From CT, FDCS and SPT are similar and sometimes it is difficult to differentiate. However, MRI demonstrates the internal architecture of the SPT better because it has the advantage of detecting hemorrhage, which is hyperintense on T1WI and hypointense on T2WI. In Liang’s report, the pancreatic FDCS appeared as hypointense on T1WI because of tumor necrosis [5]. On the other hand, the fibrosis capsule of SPT is typically hypointense on both T1WI and T2WI, which is absent in FDCS, thus increasing the likelihood of SPT diagnosis. Acinar cell carcinoma affects males more frequently than females and the patients with this disease usually have symptoms related to mass effect or metastases. It has been reported that the tumors can produce pancreatic enzymes such as lipase, amylase and elastase, which contribute to adiponecrosis, arthritis and other symptoms associated with functioning tumors [14]. Tatli et al. [17] reported that the majority of the cases were exophytic, oval or round, well-marginated masses, and were hypoenhancing on postcontrast CT and MRI. Tumors are usually solid when small but cystic degeneration can also be detected in large lesions [17]. However, the symptoms of pancreatic FDCS vary from no symptoms to paraneoplastic syndrome, as in our case. They do not present with symptoms due to production of pancreatic enzymes. From most reports, extranodal FDCS are relatively hypervascular, although some cases were hypovascular. This feature can also contribute to the differential diagnosis of the two tumors.

Paraneoplastic pemphigus is a life-threatening bullous autoimmune disease that is characterized by severe stomatitis and polymorphous skin eruptions [18]. Myasthenia gravis is reported to be a complication of PNP [19]. The pathogenesis is thought to be due to the production of antibodies against tumor-derived antigens; the antibodies may attack components sharing the same or a similar epitope [20]. To date, few cases of PNP associated with intra-abdominal FDCS have been reported, and only 1 case of PNP and MG associated with an FDCS in the axillary region and neck has been reported [21].

Our patient was the first case of PNP and MG associated with pancreatic FDCS. Cases of FDCS associated with PNP were also reported to suffer a higher degree of malignancy. Extranodal FDCS also presented with relative high aggression. Therefore, our case, an extranodal FDCS associated with PNP and MG, was highly aggressive. Although treated with surgery, the patient’s symptoms did not disappear but kept progressing. Several reasons may be responsible for this. First, since the tumor was highly malignant, it may have invaded neighboring tissues or organs before the operation. Second, a release of massive antibodies when touching the tumor during surgery would temporarily result in a more serious condition. Even after the resection of the tumor, plenty of residual antibodies also promoted the progression of the disease. Finally, the patient was in a consumption status with poor resistance associated with systemic inflammatory response to bacterial and fungal infection in the lungs. The long-term use of hormones may also have led to immune dysfunction [22].

The optimal treatment of FDCS is unclear because of the rarity of the disease. The current treatment is complete surgical resection for localized tumors, adjuvant chemotherapy and radiotherapy may be combined in cases with adverse prognostic factors.

In conclusion, pancreatic FDCS is rare, and pancreatic FDCS manifested as PNP and MG is even rarer. The CT features of the pancreatic FDCS in our case are a well-defined, solid mass with central necrosis, progressive enhancement, serpentine feeding arteries and a draining vein in the solid component of the tumor. The tumor should be differentiated from neuroendocrine tumors, solid papillary neoplasms and acinar cell carcinoma. When it is associated with PNP and MG, extranodal FDCS would become highly aggressive, so the prognosis is poor even after surgery and other intensive treatment.

## Data Availability

Data supporting the results reported in the article can be found in the database of Sichuan Provincial People’s Hospital.

## Abbreviations

AFP: Alpha-fetoprotein
ANA: Antinuclear antibody
ANCA: Anti-Neutrophil Cytoplasmic Antibodies
anti-AchR: antibody anti-acetylcholine receptor antibody
anti-CENP-B antibody: anti-centromere protein B antibody
CA125: Carbohydrate antigen125
CA199: Carbohydrate antigen125
CD117: Cluster of Differentiation117
CD138: Cluster of Differentiation138
CD21: Cluster of Differentiation21
CD23: Cluster of Differentiation23
CD34: Cluster of Differentiation34
CEA: Carcinoembryonic antigen
CK: Cytokeratin
CT: Computed tomography
Des: Desmin
DOG-1: Delay of germination 1
EBER: Epstein-Barr virus-encoded RNA
EBV: Epstein-Barr virus
FDC: Follicular dendritic cells
FDCS: Follicular dendritic cell sarcoma
MG: Myasthenia gravis
MRI: Magnectic resonace imaging
PNP: Paraneoplastic pemphigus
SMA: Antismooth muscle antibody
SPT: Solid pseudopapillary tumors
T1WI: T1 weighted imaging
T2WI: T2 weighted imaging
